# Assessment of Quality of Life and Pain Severity in Older Men with Osteoporosis: Cross-Sectional Study

**DOI:** 10.3390/ijerph182111276

**Published:** 2021-10-27

**Authors:** Agnieszka Nawrat-Szołtysik, Zuzanna Miodońska, Laura Piejko, Bogna Szołtys, Monika Błaszczyszyn, Beata Matyja, Ryszard Zarzeczny, Izabela Zając-Gawlak, Ewa Kucio, Anna Polak

**Affiliations:** 1 Institute of Physiotherapy and Health Sciences, Jerzy Kukuczka Academy of Physical Education, 40-065 Katowice, Poland; l.piejko@awf.katowice.pl (L.P.); bo.szoltys@gmail.com (B.S.); a.polak@awf.katowice.pl (A.P.); 2 Center Saint Elizabeth, 41-700 Ruda Śląska, Poland; beatamatyja@interia.pl; 3 Department of Medical Informatics and Artificial Intelligence, Faculty of Biomedical Engineering, Silesian University of Technology, 41-800 Zabrze, Poland; zuzanna.miodonska@polsl.pl; 4 Faculty of Physical Education and Physiotherapy, Opole University of Technology, 45-027 Opole, Poland; mblaszczyszyn@wp.pl; 5 Institute of Health Sciences, The Jan Kochanowski University of Kielce, 25-369 Kielce, Poland; zarzecznyryszard2@gmail.com; 6 Institute of Sport Science, Jerzy Kukuczka Academy of Physical Education, 40-065 Katowice, Poland; i.zajac-gawlak@awf.katowice.pl; 7 Department of Physiotherapy, Jerzy Kukuczka Academy of Physical Education, 40-065 Katowice, Poland; e.kucio@awf.katowice.pl; 8 American Heart of Poland, St. Elizabeth’s Hospital, 40-008 Katowice, Poland

**Keywords:** osteoporosis, older men, quality of life, mobility, pain, physical activity

## Abstract

Background: The quality of life in osteoporosis is studied for men rather than for women. Aim of the study was to determine how bone mass density (BMD) relates to life quality components and the severity of pain felt by men affected by osteoporosis. Methods: Presented research is a cross-sectional study. The cohort of 62 men aged 65 to 85 years was divided into a group with osteoporosis (N = 27) and a group without osteoporosis (N = 35). The participants’ quality of life was measured with the Qualeffo41 Questionnaire, BMD was quantified by densitometry, and pain intensity was assessed on the Visual Analogue Scale. Results: We found that lower BMD was strongly correlated to participants’ quality of life (r = −0.72), especially the quality of leisure and social activities (r = −0.66), general health perception (r = −0.59), and mobility (r = −0.57). Pain significantly affected general health perception in older men with osteoporosis. General health assessment and pain were highly correlated with each other (r = 0.888). Conclusion: BMD and the overall quality of life of the study participants were related to each other. The strongest relationship occurred between reduced BMD and leisure and social activities component. The pain significantly affected participants’ general health perception. The results may be employed to create new prophylactic strategies to improve life quality in men with osteoporosis.

## 1. Introduction

WHO defines the quality of life as an achievable optimal level of physical, mental, and intellectual abilities, roles and social functioning, perception of health, life satisfaction, and general well-being in a person with a specific disease or during the treatment process [1]. One of the chronic diseases affecting elderly people is osteoporosis, a skeletal condition reducing bone strength and increasing the risk for fragility fracture [2]. The incidence of osteoporosis is greater in women than in men, but in recent years, bone mass loss has been increasingly reported as major health problem also in the male population [3]. The WHO statistics estimate the number of patients affected by the disease at around 75 million in Europe, Japan, and the USA [1]. According to the International Osteoporosis Foundation (IOF) and the European Federation of Pharmaceutical Industry Association (EFPIA), 22 million women and 5.5 million men were estimated to have osteoporosis [4].

The central and peripheral skeletons reach their peak mass in men at the age of around 20 and 30 years, respectively. In both men and women, peak bone mass is determined by the duration of puberty (longer in men, whereby their peak bone mass is greater than women’s) and genetic factors, which altogether account for 70−85% of bone mineral density of the central skeleton and the proximal femoral head and for 50−60% of bone mineral density of the peripheral skeleton. Skeletal growth can be obstructed by a number of external factors (e.g., insufficient exercise, limited dietary calcium intake, cigarette smoking, alcohol abuse, glucocorticoid therapies), which may prevent bone mass from reaching its optimum level [5].

Factors increasing the risk of osteoporosis are similar between men and women. These are usually hypogonadism, digestive system diseases, vitamin D deficiency, anticonvulsant drug therapies, low BMI, alcohol abuse, cigarette smoking, as well as long-term treatment with some chronic disease drugs [6]. Bone mass homeostasis is also strongly influenced by changes in levels of sex hormones (androgens and estrogens) [7].

Pain associated with osteoporosis limits patients’ mobility, reduces their living space, and increases their dependence on third persons. As it intensifies, it can also lead to postural deformities and reduce physical fitness and life energy in affected men. The quality of life in patients with osteoporosis is also frequently indicated to be eroded by osteoporotic fractures. Vertebral fragility fractures are the most common type of osteoporotic fractures, occurring in about 30–50% of osteoporotic patients [8]. The number of older men reporting such traumas has increased in recent years [9,10]. It is estimated that men account for 20% of vertebral fractures and for 30% of hip fractures. For reasons that are not yet known, men die markedly more often than women from hip fractures, spinal fractures, and other major osteoporotic fractures [10,11].

Specifically designed therapeutic exercises (i.e., early mobilization exercises, strengthening, and balance training) are beneficial for subjects with bone loss, improving their functioning and reducing the risk of falling. Another non-pharmacological therapeutic option is short-term use of the spinal orthoses [12], with potential positive effects on the muscle strength of the trunk extensors [13].

Most men start losing bone mass as they turn 41. The pace of the process is relatively similar between men and women, but greater periosteal apposition in men mitigates its impacts more effectively. In addition, the production of sex hormones in ageing men decreases linearly, so they do not lose bone mass as rapidly as women do. As a result, men start experiencing osteoporotic fractures about 5–7 years later compared to women [14], but the consequences of fractures in men are greater than in women. Men have about twice the 1-year fatality rate after hip fracture compared to women [15]. Men affected by osteoporotic fractures often show disability, with a consequent low independence in activities of daily living [16]. The consequences of fractures in men also affect their quality of life [17]. The analysis of the literature done by Rinonapoli et al. [18] showed that male osteoporosis is underscreened, underdiagnosed, and undertreated, and therefore increased attention must be paid to osteoporosis in men.

Scatturo et al. [19] performed a longitudinal cohort study to evaluate the effectiveness of postural training, resistance exercises, and visual stabilization exercises on pain, postural balance, and quality of life of women suffering from osteoporosis who suffered multiple vertebral fragility fractures and took antiresorptive drugs. The combined approach reduced pain and improved patients’ quality of life.

After conducting a meta-analysis of studies investigating the quality of life in patients with osteoporosis, Crompton et al. concluded that the great majority of the available data concerned osteoporosis in women. Men with osteoporosis and their quality of life were rarely investigated [14].

### Aim

Given the above, this study was designed:

1. To determine whether, in men with osteoporosis, bone mass density and life quality components are related to each other and
2. To assess the level of pain felt by men with osteoporosis and its impact on their quality of life.

## 2. Material and Methods

### 2.1. Study Design

Qualitative descriptive study was performed to observe the phenomenon of osteoporosis in men as well as the searching and verifications of relationships between the symptoms of osteoporosis (pain) and the quality of life of the respondents.

The study protocol was approved by the Biomedical Committee at the Academy of Physical Education in Katowice (decision No.7/2009), as it followed all the required legal and ethical standards.

### 2.2. Participants

All respondents lived permanently in city care homes. The home care provides round-the-clock care, meets the needs of residents (living, health, social, religious, educational), and creates conditions for a peaceful, safe, and dignified life. The inclusion criteria were: (1) men over the age of 65, who live permanently in the home care facility; (2) the ability to function independently or with a little help from others (over 80 points according to the Barthel scale); (3) balanced mood (below 16 points according to the CES-D Scale); (4) written consent to participate in the study; and (5) understanding of the essence of the research. The exclusion criteria were: (1) men under the age of 65; (2) no permanent residence in the home care facility; (3) diseases of the nervous system (neuropathies, stroke, damage to the cerebellum, labyrinth, Parkinson’s disease); (4) no logical verbal contact with the resident; (5) inability to move independently; (6) partial or complete dependence on the performance activities of daily livings (less than 80 points according to the Barthel scale); (7) mild to moderate depression (above 16 points according to the CES-D Scale); (8) refusal to consent to participate in the study; and (9) lack of understanding of the essence of research by the resident. Ultimately, the study group comprised 62 men aged between 65 and 85 years, who all provided voluntary informed written consent to participate in the study.

### 2.3. Blinding

The main researcher set the group assignments in sequentially numbered, sealed, opaque envelopes based on the results of the bone density score. The envelopes were concealed from the medical personnel and therapists, and the main researcher took steps for blinding and managed all the residents’ data in an anonymized format. The medical personnel, therapist, and residents were blinded as to who was being assigned to each group.

### 2.4. Outcomes

#### 2.4.1. Bone Density

Bone density measurements were obtained at the distal radius using the LUNAR densitometer (PIXI, GE Medical Systems Lunar, Madison, WI, USA). We chose peripheral densitometry instead of central densitometry in order to perform the examination in the participants’ places of residence.

#### 2.4.2. Quality of Life

Participants’ quality of life was measured with the Qualeffo41 Questionnaire (the Quality of Life Questionnaire of the European Foundation for Osteoporosis). This questionnaire contains 41 questions divided into 7 components designed to assess pain (QOL1), the activities of daily living (QOL2), the ability to do jobs around the house (QOL3), mobility (QOL4), leisure and social activities (QOL5), general health perception (QOL6), and mental function (QOL7). The minimum score on the questionnaire is 0, and the maximum is 100. A higher mean score indicates a lower quality of life [20]. The validation in Polish and the assessment of psychometric properties was carried out under the supervision of Bączyk [21,22] and Lips [23] by a group of experts in the field of osteoporosis at the European Foundation for Osteoporosis. The reliability of the Qualeffo41 scale as assessed by the internal consistency coefficient (Cronbach’s α) is quite high. The Cronbach’s α internal consistency coefficient is 0.72 for the entire scale, while for individual subscales, the values range from 0.72 for general perception of health and 0.77 to 0.92 for physical functioning.

#### 2.4.3. Pain

The presence of pain and its intensity were assessed on the Visual Analogue Scale (VAS), where 0 is no pain at all and 10 the worst pain imaginable [24].

### 2.5. Statistical Analysis

Collected dataset was subjected to statistical analysis. The normality of distributions was assessed using the Shapiro–Wilk test. Because most of the variables had non-normal distributions, analysis was continued with the Mann–Whitney–Wilcoxon rank-sum test (MWW) and Spearman’s rank correlations. The results were deemed statistically significant at *p* < 0.05.

## 3. Results

The study was conducted from January to December, 2018. The study participants were the residents of two care homes in the Silesian Voivodeship in Poland. The residents were people with health indications (significant loss of psychophysical fitness, advanced old age) who were deprived of the care of relatives. The residents had low level of daily physical activity. Due to staying in a care home facility, all respondents had a homogeneous, balanced diet and a proper nutritional status. From 90 men assessed for eligibility, 28 were excluded from the study (mostly because of neurological disorders or low results on the Barthel scale).

Participants with T-score ≤ −2.5 in were assigned to Group 1 (osteoporosis) and those with T-score > −2.5 (without osteoporosis) to Group 2. Group 1 consisted of 27 men with a median T-score of −3.1, a median age of 79 years, and a median BMI of 27.1. The median T-score, age, and BMI of 35 men in Group 2 were −1.9, 71 years, and 25.86, respectively (Table 1).

The baseline characteristics and quality-of-life scores were compared between the groups using MWW test. The test showed that the groups were not significantly different in terms of age (*p* = 0.051) or BMI (*p* = 0.081). Significant between-group differences for overall quality of life (*p* = 0.001) as well as for some of its components, i.e., QOL4 (*p* = 0.011), QOL5 (*p* ≤ 0.001), and QOL6 (*p* = 0.003) (Table 2).

**Figure 1:**
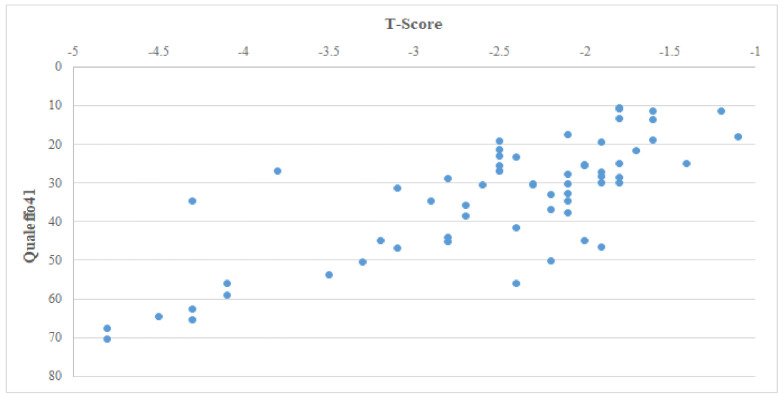
Scatter plot of participants’ bone density (T-score) and quality of life (Qualeffo41). The distribution of data points corresponds to a strong correlation between these measures.

Statistically significant correlations were determined between reduced bone density and participants’ scores on the quality of life components. The correlations were moderately strong for QOL5, QOL6, and QOL4 and weak for QOL1 and QOL7 (Table 3). The participants with lower bone density had higher scores on the Qualeffo41 scale, pointing to a strong correlation between bone density and quality of life (r = −0.72; Figure 1).

**Figure 2:**
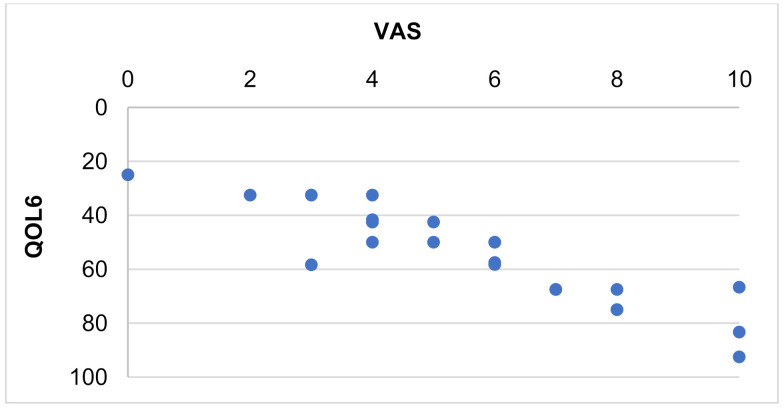
Scatter plot of pain severity (VAS) and general health perception (QOL6) in men with osteoporosis.

The correlations between the pain scores of participants in Group 1 and the quality of life showed that neither the overall quality of their lives nor most of its components were significantly affected by pain; the exception was QOL6 (general health perception) (Table 4, Figure 2).

## 4. Discussion

Quality of life is a multifaceted construct influenced by the levels of happiness, physical and mental well-being, and satisfaction with life as well as a sense of fulfilling one’s life aspirations and plans. It is adversely affected by deteriorating health, a loss of self-esteem, sleeping disorders, mobility problems, more frequent pain sensations, and chronic conditions, such as osteoporosis. It has been shown that persons affected by a disease, especially a chronic one, have a lower quality of life than healthy, same-age persons. The very presence of such a disease is sufficient to reduce satisfaction with life [25]. Similarly, in the presented study, the quality of life of men with osteoporosis measured on the Qualleffo41 scale was significantly lower (a median of 38 pts) compared with the healthy participants (28 pts). According to Beauvais et al. [26], men affected by osteoporosis are poor at coping with a loss of function. Moriyama et al. and Ferrier et al. observed that osteoporosis makes people pessimistic, depresses their mood, and results in sleeplessness and fatigue [20,27].

Obtained results are even more important, as they concern men living in care facilities, which may affect quality of life independently of chronic diseases. Grzegorczyk et al. [16] indicated that satisfaction with life depends not only on the biological state of a person but also on their personality and milieu. A meta-analysis of studies led authors to a conclusion that elderly people living in care homes had lower quality of life than those living with their families or attending the Universities of the Third Age (U3A). After assessing 128 residents of care homes and 45 UTA students (mean age 71.5 years) on the Nottingham Health Profile (NHP), the same authors reported that UTA students scored significantly higher on most items except for pain and sleep quality [28].

Szczuka et al. observed that, paradoxically, the quality of life of persons with osteoporosis may improve after the diagnosis, especially when the disease becomes a strong motivator for them to shed unhealthy habits and adopt a sustainable style of living [29].

While osteoporosis reduces quality of life, it does not affect all its components [30,31]. In this study, participants with osteoporosis gave the lowest scores to QOL5 (leisure and social activities), QOL6 (general perception of health), and QOL4 (mobility) and the highest to QOL1 (pain) and QOL2 (the activities of daily living). A probable reason why relatively few participants reported difficulty with cleaning, cooking, washing up, and shopping was that all these activities were done for them by the care homes staff. A meta-analysis of studies led Al-Sari et al. to conclude that lower quality of life of persons with osteoporosis reveals itself more through physical function than mental function [32]. The observation was then confirmed by other authors. Brennan-Olsen et al. reported from a cross-sectional study of 893 Australian men aged 24–92 years that a limited ability to perform daily tasks had a strong negative influence on the quality of life in all of them regardless of their age and the intensity of anxiety and depression symptoms [33]. The findings of our study are somewhat different from what other authors report because the correlations between pain and the overall quality of our participants’ lives as well as most of its components were not statistically significant. The only quality-of-life component that was strongly correlated with pain was general health perception (QOL6, r = 0.888). The differences may be due to the fact that our exclusion criteria prevented the participation of men with recent osteoporotic fractures causing sudden, sharp, and strong pain. Osteoporosis is a generalized skeletal disease that increases the risk of bone fractures causing chronic pain and reducing physical fitness [34,35,36]. Ostrowska et al. [37] observed that in many patients, these symptoms are associated with psycho-social discomfort eroding their quality of life. Leidig-Bruckner et al. concluded that people who feel pain are likely to give lower ratings to the quality of their lives [38]. They also found that the quality of life reduction caused by spinal pain is comparable whether caused by osteoporosis or osteoarthritis. It is also reported that pain caused by a vertebral fracture is one of the main factors affecting the quality of life because of its impact on sleep quality, emotional health, and mobility [10,39,40]. Presented research showed that reduced bone density affects specific components of quality of life in men. According to our knowledge, no works showing this kind of analysis for Polish older men have been reported before. The results may be employed to create new, prophylactic strategies aimed at improving life quality in men with osteoporosis. These strategies should consider, i.a., education on activities of entertainment and social activity that are adjusted to osteoporosis, which could lead to improved mobility and general health perception in patients. In the future, it is planned to extend the research to a larger number of subjects, including men living in independent households, to verify whether living in a care facility has any impact on the findings.

### Study Limitation

The low number of participants of this study and monocentric design does not allow to draw strong conclusions. Test results should be confirmed on high-quality therapeutic experiments on a larger number of subjects from different facilities with control groups.

## 5. Conclusions

Reduced bone mass was significantly correlated with both the overall quality of life and its components in all study participants. The strongest significant correlations were found for leisure and social activity, the general perception of health, and mobility and the weakest but still significant for pain intensity and mental function. Pain did not significantly affect the overall quality of participants’ lives. A significant influence of pain was only observed in participants’ self-ratings of health. However, it should be noted that the low number of participants in this study does not allow strong conclusions to be drawn. The results should be confirmed on a larger group of patients from different care facilities in Poland.

### Highlights

− At present, the scientific literature does not provide sufficient, high-quality research and reports on the quality of life in older men with osteoporosis.
− We studied the relationship between bone density and components of quality of life.
− We assessed pain, quality of life, and bone density in 62 older men.
− Bone mass density was related to the quality of life, mostly leisure and mobility.
− Pain significantly affects general health perception in older men with osteoporosis.

## Figures and Tables

**Table 1:** Participants’ age, body mass index (BMI), and T-scores.

	Group 1 (Osteoporosis)				Group 2 (without Osteoporosis)			
	Median	IQR	Max	Min	Median	IQR	Max	Min
Age	79	15	85	65	71	6.75	80	65
BMI	27.10	6.99	29.03	23.34	25.86	1.65	36.79	19.29
T-score	−3.10	1.48	−4.3	−2.5	−1.90	0.30	−2.4	−1.4

**Table 2:** Between-group differences in the overall quality of life and its components.

	Group 1 (Osteoporosis)		Group 2 (No Osteoporosis)		MWW
	Median	IQR	Median	IQR	*p*
QOL1	25	38	20	15	0.084
QOL2	25	34	20	20	0.107
QOL3	40	53	30	28	0.059
QOL4	50	47	25	26	**0.011**
QOL5	58	32	30	23	**<0.001**
QOL6	50	25	42	21	**0.003**
QOL7	43	29	33	24	0.111
Qualeffo41	38	27	28	14	**0.001**

Statistically significant results of Mann–Whitney–Wilcoxon test (*p* < 0.05) are bolded.

**Table 3:** Correlations between participants’ bone density and quality of life.

	Spearman’s Correlation	
	rho	*p*
QOL1	−0.36	**0.004**
QOL2	−0.44	**<0.001**
QOL3	−0.46	**<0.001**
QOL4	−0.57	**<0.001**
QOL5	−0.66	**<0.001**
QOL6	−0.59	**<0.001**
QOL7	−0.39	**0.002**
Qualeffo41	−0.72	**<0.001**

Statistically significant results of Spearman’s correlation coefficient (*p* < 0.05) are bolded.

**Table 4:** Correlations between pain intensity and the quality of life in Group 1 (participants with osteoporosis).

	Spearman’s Correlation	
	rho	*p*
Pain (QOL1)	0.179	0.371
Activities of daily living (QOL2)	0.031	0.879
Jobs around the house (QOL3)	0.025	0.903
Mobility (QOL4)	0.324	0.099
Leisure, social activities (QOL5)	0.089	0.659
General health perception (QOL6)	0.888	**<0.001**
Mental function (QOL7)	0.305	0.121
Qualeffo41	0.284	0.152

Statistically significant results of Spearman’s correlation coefficient (*p* < 0.05) are bolded.

## Data Availability

The data presented in this study are available on request from the corresponding author.
